# The effect of acute and chronic sprint‐interval training on LRP130, SIRT3, and PGC‐1*α* expression in human skeletal muscle

**DOI:** 10.14814/phy2.12879

**Published:** 2016-09-07

**Authors:** Brittany A. Edgett, Jacob T. Bonafiglia, Brittany L. Baechler, Joe Quadrilatero, Brendon J. Gurd

**Affiliations:** ^1^ School of Kinesiology and Health Studies Queen's University Kingston ON Canada; ^2^ Department of Kinesiology University of Waterloo Waterloo ON Canada

**Keywords:** Exercise, LRP130, LRPPRC, PGC‐1*α*, SIRT3, skeletal muscle

## Abstract

This study examined changes in LRP130 gene and protein expression in response to an acute bout of sprint‐interval training (SIT) and 6 weeks of SIT in human skeletal muscle. In addition, we investigated the relationships between changes in LRP130, SIRT3, and PGC‐1*α* gene or protein expression. Fourteen recreationally active men (age: 22.0 ± 2.4 years) performed a single bout of SIT (eight, 20‐sec intervals at ~170% of VO_2_peak work rate, separated by 10 sec of rest). Muscle biopsies were obtained at rest (PRE) and 3 h post‐exercise. The same participants then underwent a 6 week SIT program with biopsies after 2 (MID) and 6 (POST) weeks of training. In response to an acute bout of SIT, PGC‐1*α* mRNA expression increased (284%, *P *<* *0.001); however, LRP130 and SIRT3 remained unchanged. VO_2_peak and fiber‐specific SDH activity increased in response to training (*P *<* *0.01). LRP130, SIRT3, and PGC‐1*α* protein expression were also unaltered following 2 and 6 weeks of SIT. There were no significant correlations between LRP130, SIRT3, or PGC‐1*α* mRNA expression in response to acute SIT. However, changes in protein expression of LRP130, SIRT3, and PGC‐1*α* were positively correlated at several time points with large effect sizes, which suggest that the regulation of these proteins may be coordinated in human skeletal muscle. Future studies should investigate other exercise protocols known to increase PGC‐1*α* and SIRT3 protein, like longer duration steady‐state exercise, to identify if LRP130 expression can be altered in response to exercise.

## Introduction

Leucine‐rich pentatricopeptide repeat containing (LRPPRC, aka LRP130) is a novel transcriptional coactivator proposed to promote the expression of mitochondrial‐encoded genes, resulting in increases in oxidative phosphorylation and fatty acid oxidation (Cooper et al. 2006, 2008; Gohil et al. 2010; Sasarman et al. 2010; Liu et al. 2011). While the mechanisms underlying this response are unknown, one proposed mediator is the transcriptional coactivator peroxisome proliferator activated receptor gamma coactivator 1 alpha (PGC‐1*α* aka PPARGC1Α) (Cooper et al. 2008). In the liver, LRP130 forms part of the PGC‐1*α* holocomplex, and regulates gluconeogenic and mitochondrial gene expression (Cooper et al. 2006). Additionally, LRP130 is deacetylated, and subsequently activated in the liver by the mitochondrial NAD^+^‐dependant deacetylase sirtuin 3 (SIRT3), resulting in increased expression of oxidative phosphorylation genes (Liu et al. 2014). Changes in LRP130 expression are also potentially involved in regulating LRP130 activity, and several studies have implicated PGC‐1*α* in the control of LRP130 and SIRT3 expression (Cooper et al. 2008; Kong et al. 2010; Giralt et al. 2011; Brandauer et al. 2015), and vice versa (Shi et al. 2005; Cooper et al. 2006, 2008; Palacios et al. 2009; Liu et al. 2014). At present, the regulation of LRP130 in human skeletal muscle has not been examined, and whether there are relationships between changes in LRP130 with SIRT3 or PGC‐1*α* expression is unknown.

Increases in skeletal muscle mitochondrial gene and protein expression are typical responses to aerobic exercise training (Hood 2001; Perry et al. 2010; Booth and Neufer 2012), promoting increases in fatty acid oxidation and oxidative phosphorylation (Holloszy 2008). These adaptations are proposed to be mediated in part by PGC‐1*α* and SIRT3; not only due to increases in their activation, but also their protein abundance in response to aerobic exercise training (Holloszy 2008; Palacios et al. 2009; Perry et al. 2010; Vargas‐Ortiz et al. 2015). It is well established that acute aerobic exercise increases *pgc‐1α* mRNA expression (Pilegaard et al. 2003; Perry et al. 2010; Edgett et al. 2013), and training induces increases in PGC‐1*α* protein and mitochondrial proteins (Burgomaster et al. 2008; Perry et al. 2010; Ma et al. 2013). Similarly, SIRT3 protein is elevated in human skeletal muscle following aerobic exercise training (Johnson et al. 2015; Vargas‐Ortiz et al. 2015). In rat skeletal muscle, LRP130 gene and protein expression are elevated in response to swimming for 7 days (Vechetti‐Junior et al. 2016); however, to date, no studies have investigated the effect of exercise on LRP130 in human skeletal muscle. Thus, while PGC‐1*α*, SIRT3, and mitochondrial gene and/or protein expression increase following acute and chronic aerobic exercise (Perry et al. 2010; Ma et al. 2013; Johnson et al. 2015; Vargas‐Ortiz et al. 2015), whether LRP130 expression is altered in response to exercise in human skeletal muscle is currently unknown.

Given that SIRT3 and PGC‐1*α* are proposed regulators of LRP130 expression and activity, and both are upregulated in response to aerobic exercise in human skeletal muscle, it is reasonable to speculate that LRP130 expression should increase in concert with SIRT3 and PGC‐1*α* following both acute and chronic aerobic exercise. Importantly, upregulation of LRP130 expression could play a role in mediating training‐induced increases in skeletal muscle mitochondrial proteins and oxidative capacity by increasing the transcription of mitochondrial genes. Therefore, the primary purpose of this study was to examine changes in LRP130 gene and protein expression in response to a single acute bout, and 6 weeks of sprint‐interval training (SIT), respectively. In addition, we examined whether a relationship exists between changes in SIRT3, LRP130, and PGC‐1*α* gene or protein expression following acute and chronic SIT in human skeletal muscle.

## Materials and Methods

### Ethics statement

All participants were young, healthy, and recreationally active men (participant characteristics are presented in Table 1). No participant was involved in more than 3 h of aerobic exercise (running, jogging, etc.) per week or involved in any structured training program within the past 6 months. All experimental procedures were approved by the Health Sciences Human Research Ethics Board at the Queen's University and conformed to the Declaration of Helsinki. Verbal and written explanation of the experimental protocol and associated risks was provided to all participants prior to obtaining written informed consent.

**Table 1:** Participant baseline characteristics (*n *= 14)

Age (years)	22.0 ± 2.4
Height (cm)	177.8 ± 6.1
Weight (kg)	85.9 ± 17.6
BMI (kg m^−2^)	27.1 ± 5.4
Absolute VO_2_peak (mL min^−1^)	3375 ± 615
Relative VO_2_peak (mL min^−1^ kg^−1^)	41.6 ± 7.3

Values are mean ± standard deviation.

### Experimental design

**Figure 1:**
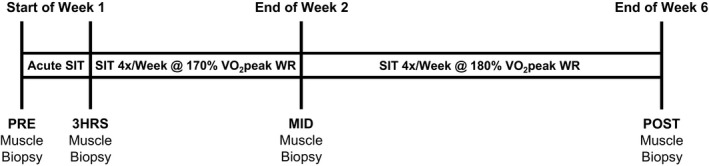
Study protocol overview.

This study involved the completion of one intervention with two distinct parts. The first examined the effect of acute SIT on the mRNA expression of *pgc‐1α*,* sirt3*, and *lrp130*. The second utilized the same participants to examine the effect of 6 weeks of SIT on the protein abundance of PGC‐1*α*, SIRT3, and LRP130. All physiological testing and training was performed on a Monark Ergomedic 874 E stationary ergometer (Vansbro, Sweden). Participants were asked to refrain from alcohol and exercise 24 h before, and caffeine 12 h before, all experimental visits in part one, as well as pre‐ and post‐training testing visits in part two. VO_2_peak was determined during a ramp protocol to volitional fatigue as described previously (Edgett et al. 2013), and skeletal muscle biopsy samples were obtained from the vastus lateralis using the Bergstrom muscle biopsy technique (Bergstrom 1975) modified with manual suction (Edgett et al. 2013). One portion of each muscle biopsy was embedded in Tissue‐Tek^®^ O.C.T. Compound (#4583, Sakura Finetek, St, Torrance, CA) and frozen in liquid nitrogen‐cooled isopentane for histochemical analysis. Remaining tissue was immediately frozen in liquid nitrogen and stored at −80°C until analysis by real‐time PCR and western blotting. The experimental protocol is presented in Figure 1.

### Acute exercise visit (Part 1)

Participants reported to the lab in the morning following an overnight fast (~12 h) after consuming a standardized dinner the night before [Stouffer's Sauté Sensations Country Beef Pot Roast (540 kcal; 56 g carbohydrate (CHO), 20 g fat, 14 g protein, Dole Fruit Crisp (160 kcal; 29 g CHO, 3.5 g fat, 2 g protein), and 500 mL of 2% milk (260 kcal; 24 g CHO, 10 g fat, 18 g protein)]. Upon arrival, participants were fed breakfast consisting of a plain bagel (190 kcal; 1 g fat, 36 g CHO, 7 g protein) with peanut butter (110 kcal; 10 g fat, 4 g CHO, 4 g protein) and 200 mL of apple juice (90 kcal; 22 g CHO, 0 g fat, 0 g protein). One hour after participants completed breakfast, a resting muscle biopsy (PRE) was taken. Immediately following the resting muscle biopsy, participants completed eight, 20‐sec intervals at ~170% of VO_2_peak work rate separated by 10 sec of rest for a total of 4 min, as described previously (Scribbans et al. 2014). During rest periods between intervals, participants cycled against no load at a cadence of their choice. Participants then rested for 3 h before a second muscle biopsy sample was taken (3HR) from a separate incision site on the same leg as the first biopsy (~2 cm apart).

### Training intervention (Part 2)

All participants completed training 4 days a week for 6 weeks (week 3 only had two training sessions due to a mid‐training VO_2_peak test for a total of 22 sessions). Before each training session, participants performed a warm‐up of load‐less cycling at a cadence of their choosing for 1 min. The training protocol was the same as the acute exercise prescribed in part 1 (see above for description). A mid‐training (MID) muscle biopsy was taken ~72 h (range: 65–72 h) following 2 weeks (8 sessions) of SIT. The mid‐training VO_2_peak test was completed ~24 h following the MID biopsy. Participants then completed the remainder of the 6 week SIT protocol at ~180% of baseline VO_2_peak work rate. Approximately 72 h (range: 67–74 h) after the last training session, a post‐training (POST) biopsy was taken, and ~24 h later, a post‐training VO_2_peak test. Both MID and POST biopsies followed the same protocol as the PRE biopsy outlined in part 1.

### RNA extraction and real‐time PCR

RNA extraction and real‐time PCR were performed as we have done previously (Edgett et al. 2013) on PRE and 3 h samples. RNA was extracted using a modified version of the single‐step method by guanidinium thiocyanate‐phenol‐chloroform extraction (Chomcyzynski and Sacchi 1987), and then quantified spectrophotometrically at 260 nm using a Take3 Plate (Biotek, Winooski, VT). Protein contamination was assessed by measuring absorbance at 280 nm (samples had an average 260:280 ratio of 1.99 ± 0.02, mean ± SD). One microgram of resulting RNA was reverse transcribed using the QuantiTect Reverse Transcription Kit (Qiagen, Mississauga, ON, Canada). Transcript levels were determined on an ABI 7500 Real Time PCR System (Foster City, CA). Primer set efficiencies were determined using real‐time PCR with an appropriate cDNA dilution series prior to sample analysis. Average primer set‐specific efficiencies (Rasmussen 2001) were *E *=* *2.00 ± 0.03 (mean ± SD). All samples were run in duplicate 25 *μ*L reactions containing: 50 ng cDNA, 0.58 *μ*mol/L primers, and GoTaq PCR Master Mix containing SYBR Green (Promega, Madison, WI). Primer sequences are provided in Table 2. All RNA data are expressed relative to TATA‐binding protein (TBP), which was stable across all states with no difference in the raw *C*_T_ values observed between resting and 3 h post‐exercise (PRE: 22.45 ± 0.40, 3 h: 22.51 ± 0.31, *P *=* *0.621).

**Table 2:** List of primer sequences used for real‐time PCR

Gene	Forward primer (5′‐3′)	Reverse primer (5′‐3′)
*pgc‐1α*	CACTTACAAGCCAAACCAACAAC	CAATAGTCTTGTTCTCAAATGGGGA
*sirt3*	GCTTCCTCTAGTGACACTGTTAG	TGCAGAAGTAGCAGTTCAGTG
*lrp130*	TTAATGATACCTGCCGCTCAG	AGCTTTAGTTCAGGCAAGAGAG
*tbp*	AGACGAGTTCCAGCGCAAGG	GCGTAAGGTGGCAGGCTGTT

### Western blotting

Samples were quantified spectrophotometrically to determine protein content (BCA Protein Assay Kit 23225, Pierce, Rockford, IL), then solubilized in sample buffer (12.5% sucrose, 1.9% SDS, 15.6 mmol/L Tris HCl pH 6.8, 0.5 mmol/L EDTA, 0.8% DTT, 0.003% bromophenol blue) and heated to 95°C for 5 min. Equal amounts of protein (7.5 *μ*g for LRP130 and PGC‐1*α* and 15 *μ*g for SIRT3) were loaded onto 12% polyacrylamide gels and separated by SDS‐PAGE. Gels were subsequently transferred to a polyvinylidene difluoride membrane via wet transfer for 1 h at 100 volts at 4°C. Membranes were blocked at room temperature by incubating in 5% BSA with Tris‐buffered saline (20 mmol/L Tris Base, 137 mmol/L NaCl, pH 7.5, adjusted using hydrochloric acid) and 0.1% Tween‐20 (TBS‐T). Blots were then incubated with primary antibodies overnight at 4°C in 5% BSA with TBS‐T. Commercially available antibodies were used to detect SIRT3 (Cell Signaling, Danvers, MA; #4590, lot# 2), LRP130 (Abcam, Cambridge, UK; ab97505, lot# GR40843‐18), and PGC‐1*α* (EMD Millipore, Darmstadt, Germany; AB3242, lot# J0510). All primary antibodies were used at a concentration of 1:1000 and diluted in 5% BSA with TBS‐T. Removal of excess primary antibody was carried out by washing the membranes in TBS‐T three times for 7 min each. The secondary antibody anti‐rabbit (#31460, Thermo Fisher Scientific, Rockford, IL) was diluted (1:30000, LRP130; 1:10000, PGC‐1*α*; 1:10000, SIRT3) and incubated with the membrane in TBS‐T (0.1%) for 1 h at room temperature. Excess secondary antibody was removed by washing the membranes in TBS‐T (0.1%) three times for 7 min each. Membranes were exposed to Immobilon Western Chemiluminescent HRP Substrate (WBKLS0500, EMD Millipore) for 5 min at room temperature and then visualized using a FluorChem HD2 system (Protein Simple, San Jose, CA). Band intensities were semiquantified using AlphaView software (Protein Simple). Amido black staining was used as a loading control and did not differ between PRE, MID, or POST samples.

### Immunofluorescent and histochemical analysis

As a general indicator of oxidative capacity, histochemical staining for succinate dehydrogenase (SDH) activity (Blanco et al. 1988) was performed as described previously (Bloemberg and Quadrilatero 2012; Scribbans et al. 2014). Briefly, sections were imaged using a Brightfield Nikon microscope linked to a PixeLink digital camera. Individual images were taken across the entire muscle cross‐section and assembled into a composite panoramic using Microsoft Image Composite Editor (Microsoft, Redmond, WA). To analyze SDH activity, compiled images were matched to corresponding fiber‐type images and ~ 30 of each fiber type were randomly selected and analyzed in Image J by subtracting background staining. Fiber types were identified via immunofluorescent analysis as described previously (Bloemberg and Quadrilatero 2012; Scribbans et al. 2014), and were categorized as type I, type IIA, or type IIX (pure IIX and hybrid IIAX). Briefly, myosin heavy chain isoforms were analyzed using primary antibodies from Developmental Studies Hybridoma Bank (Iowa City, IA) against myosin heavy chain MHC I (BA‐F8), MHC IIA (SC‐71), and MHC IIX (6H1), followed by isotype‐specific fluorescent secondary antibodies. Sections were mounted with Prolong Gold Antifade Reagent (Life Technologies, Burlington, ON, CA) and imaged the following day. These sections were visualized with an Axio Observer Z1 microscope (Carl Zeiss, Jena, TH, DE). Images were taken across entire muscle cross‐section and assembled into a composite panoramic image using AxioVision software (Carl Zeiss). SDH activity was analyzed only in type I and type IIA muscle fibers as ~64% of sections did not contain any type IIX or IIAX fibers. Data were expressed relative to the PRE values obtained, which were assigned a reference value of 1.0, and reported as mean optical density in arbitrary units.

### Statistical analysis

Statistical analysis of gene expression in part one of the intervention (acute SIT) was performed on linear data using the 2^−∆CT^ method using TBP as a housekeeping gene (Schmittgen and Livak 2008). These linear data were then compared using a paired Student's *t*‐test with statistical significance set at *P *<* *0.05. For part two (chronic SIT), a one‐way repeated measures analysis of variance (ANOVA) was used to examine the effect of time on protein expression and VO_2_peak. SDH activity was analyzed using a two‐way repeated measures ANOVA (fiber type × time). Any significant interactions or main effects were subsequently analyzed using a Bonferroni post hoc test. Correlations were performed to determine if changes in LRP130, SIRT3, or PGC‐1*α* gene and protein expression occur in association with each other in response to both acute and chronic SIT. Pearson correlation coefficient (*r*) effect sizes were classified as small (*r *= ±0.1), medium (*r *= ±0.3), or large (*r *= ±0.5) (Field 2013).

## Results

### Responses to acute exercise

**Figure 2:**
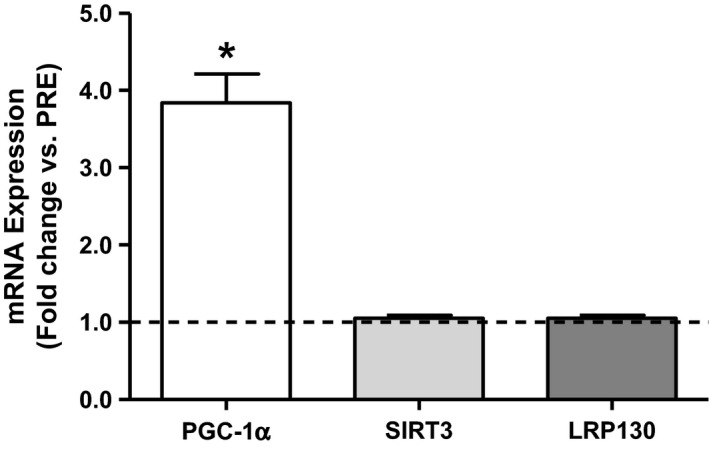
Effect of acute sprint‐interval training on skeletal muscle mRNA expression of *lrp130*,* sirt3*, and *pgc‐1α* at 3 h post‐exercise. *P < 0.0001 3HRS versus PRE. *n *= 14

In response to an acute bout of SIT, *pgc‐1α* (all gene names referring to mRNA transcripts in this article are italicized) mRNA expression increased 284% (*P *<* *0.001) 3 h post‐exercise; however, *lrp130* and *sirt3* were unaltered at this time point (Fig. 2).

### Responses to exercise training

**Figure 3:**
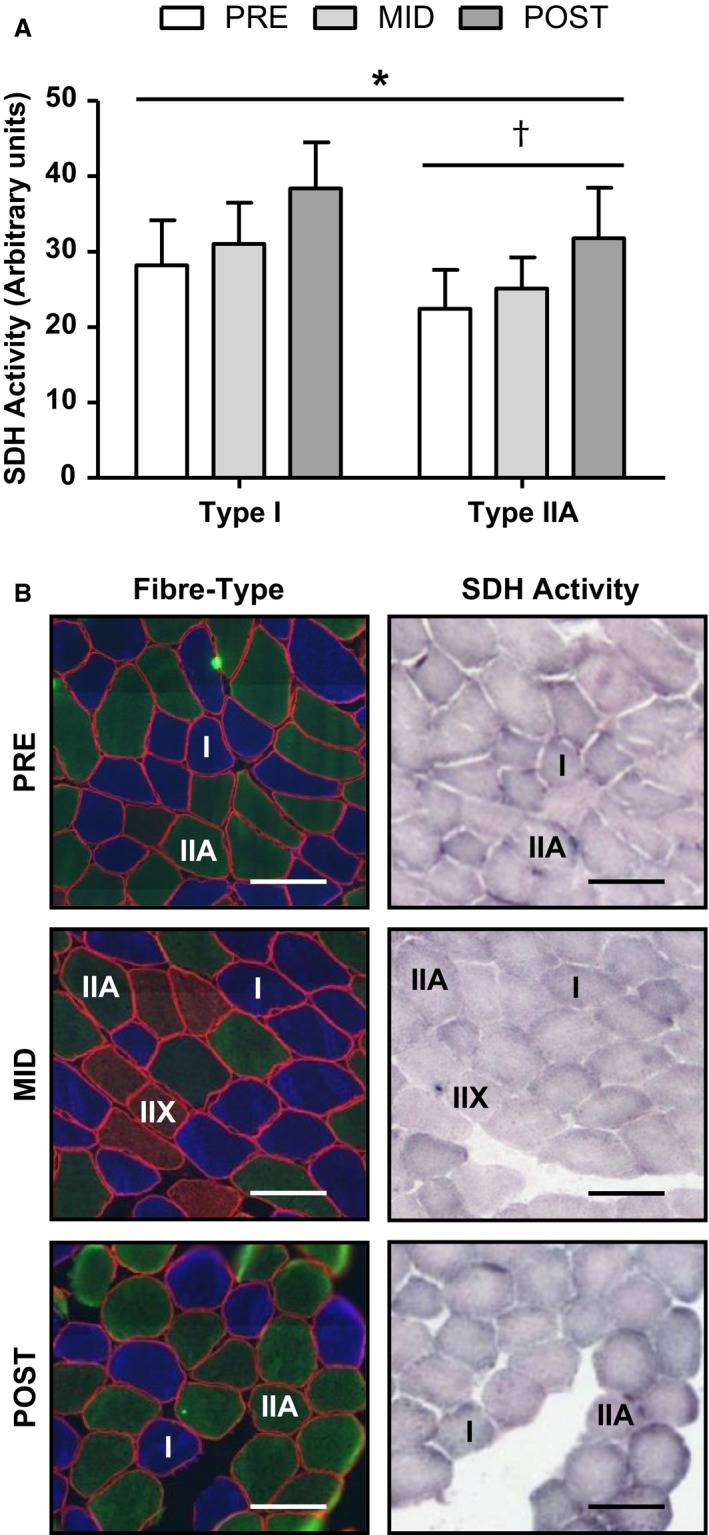
Effect of 6 weeks of sprint‐interval training on skeletal muscle succinate dehydrogenase (SDH) activity (A). Representative slides of immunofluorescent fiber‐type analysis (blue fibers are type I, green are type IIA, red/red‐green are type IIX) with serial sections of SDH activity PRE, MID, and POST (B). Values presented as mean ± SEM. Scale bars represent 100 microns (*μ*m). *Significant main effect of time, ^†^Significant main effect of fiber type. *n *= 12

A main effect of time on VO_2_peak was observed following training (mean ± SD, PRE: 3370 ± 634, MID: 3461 ± 622, POST: 3696 ± 507 mL min^−1^, *n *=* *12, *P *<* *0.01). Post hoc analysis revealed a significant increase from PRE to POST and MID to POST (*P *<* *0.05). Main effects of time (mean ± SD, PRE: 25.3 ± 6.2, MID: 28.1 ± 5.7, POST: 35.1 ± 7.1 arbitrary units, *P *<* *0.001; *n *=* *12) and fiber‐type (mean ± SD, type I: 32.5 ± 7.2, type IIA: 26.4 ± 6.6, arbitrary units, *P *<* *0.001; *n *=* *12) were observed for SDH activity in response to training (Fig. 3).

**Figure 4:**
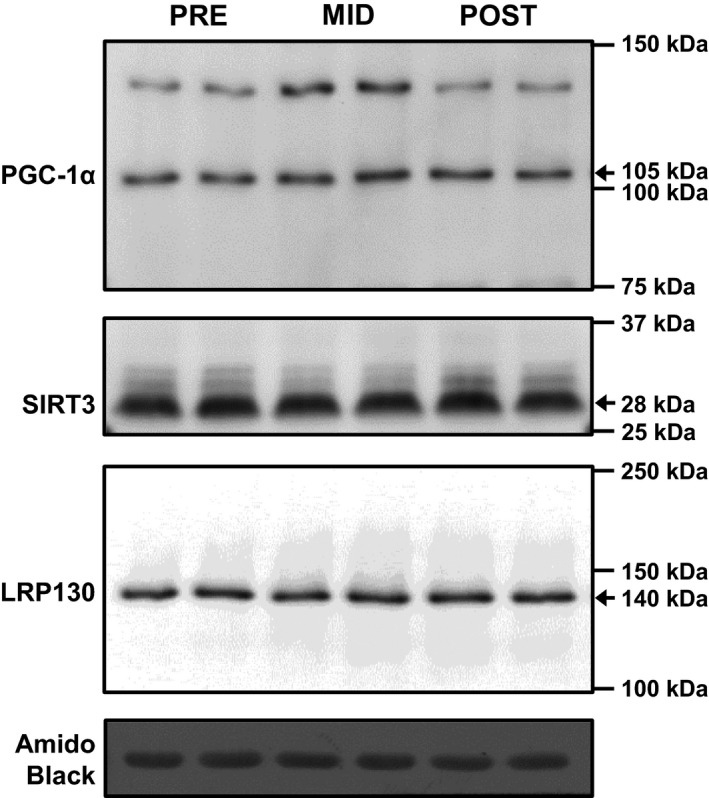
Representative blots for PGC‐1*α*, SIRT3, and LRP130 at pre‐, mid‐, and post‐training.

**Figure 5:**
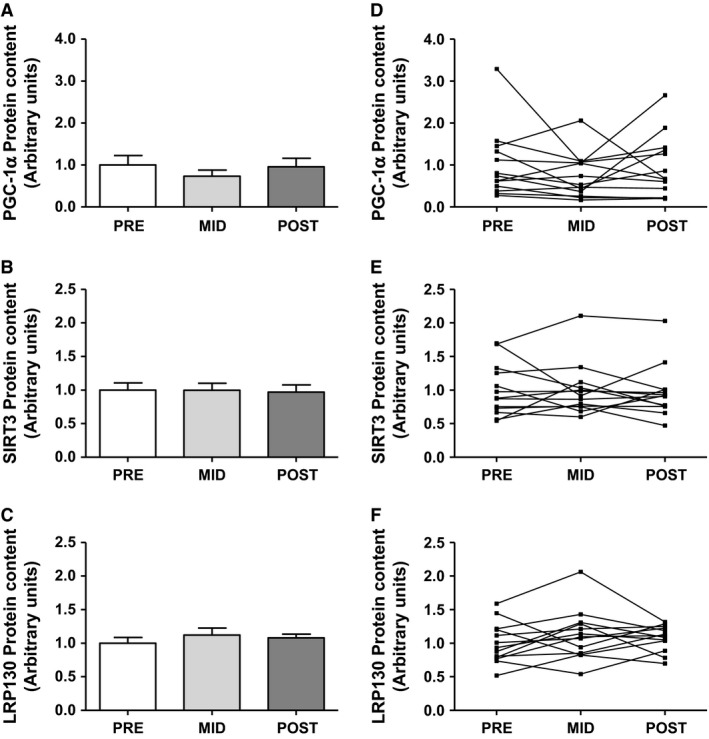
The effect of 6 weeks of SIT on the protein content of PGC‐1*α* (A, D), SIRT3 (B, E), and LRP130 (C, F) in whole muscle lysates from the vastus lateralis. Data are presented as mean ± SEM (A, B, C) and also as before and after plots depicting raw participant values (D, E, F). *n *= 13

Representative blots for LRP130, SIRT3, and PGC‐1*α* are presented in Figure 4. There was no effect of time on LRP130 (mean ± SD, PRE: 1.00 ± 0.30, MID: 1.12 ± 0.37, POST: 1.08 ± 0.19 arbitrary units, *n *=* *13), SIRT3 (mean ± SD, PRE: 1.00 ± 0.39, MID: 1.00 ± 0.39, POST: 0.97 ± 0.39 arbitrary units, *n *=* *13), or PGC‐1*α* protein expression (mean ± SD, PRE: 1.00 ± 0.81, MID: 0.73 ± 0.53, POST: 0.96 ± 0.74 arbitrary units, *n *=* *13; Fig. 5).

### Relationships between LRP130, SIRT3, and PGC‐1α expression

**Figure 6:**
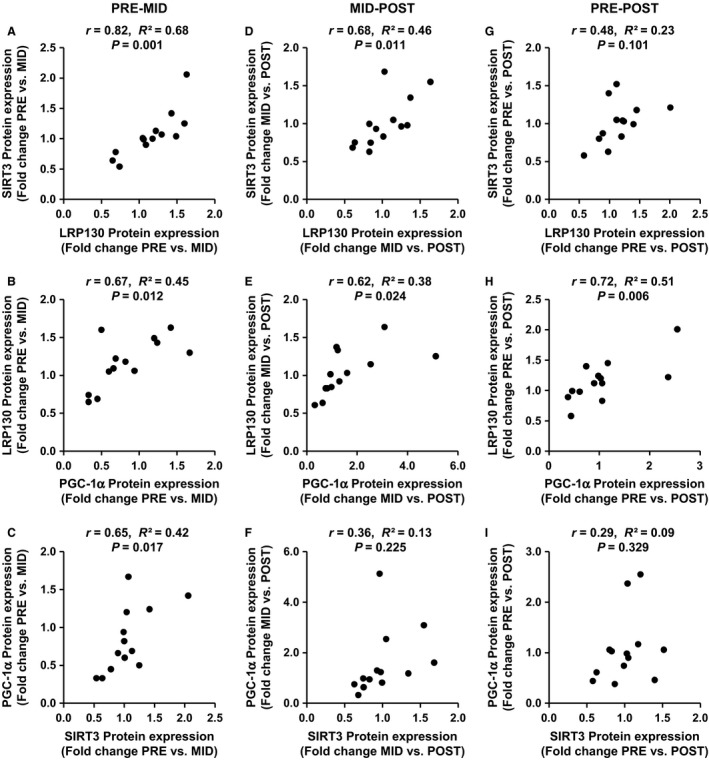
Correlations between changes in protein expression of LRP130 and SIRT3 (A, D, G), PGC‐1*α* and LRP130, (B, E, H), and SIRT3 and PGC‐1*α* (C, F, I), from PRE to MID (A, B, C), MID to POST (D, E, F), and PRE to POST (G, H, I). *r*, Pearson's correlation coefficient; *R* ^2^, coefficient of determination*. n *= 13

Correlations of individual changes in LRP130, SIRT3, and PGC‐1*α* mRNA or protein expression are presented in Table 3 and Figure 6, respectively. There were no significant correlations between *lrp130*,* sirt3*, or *pgc‐1α* mRNA expression in response to an acute bout of SIT. However, correlations between changes in protein expression of LRP130, SIRT3, and PGC‐1*α* from PRE to MID were significant. From MID to POST, only LRP130 was related to changes in SIRT3 and PGC‐1*α*, while from PRE to POST, only LRP130 was significantly related to changes in PGC‐1*α* protein expression. Effect sizes were large for all significant correlations, and all variables were positively related.

**Table 3:** Correlations among changes in mRNA expression of *lrp130*,* sirt3*, and *pgc‐1α*

	*sirt3*	*pgc‐1α*	Group mean change	SD
*lrp130*	*r *=* *0.31, *P *=* *0.287	*r *=* *0.04, *P *= 0.883	5%	14%
*sirt3*	–	*r *=* *0.11, *P *= 0.717	5%	13%
*pgc‐1α*	–	–	284%	140%

SD, standard deviation; *n *= 14.

## Discussion

LRP130 is a novel transcription cofactor proposed to regulate the expression of mitochondrial‐encoded genes via its interactions with the mitochondrial deacetylase SIRT3 (Liu et al. 2014) and the transcriptional coactivator PGC‐1*α* (Cooper et al. 2006, 2008). This study is the first to investigate whether LRP130 gene and protein expression are altered in response to an acute bout, and 6 weeks of SIT, respectively. In addition, we examined whether there were any relationships between changes in LRP130, PGC‐1*α*, and SIRT3 gene or protein expression following acute and chronic SIT in human skeletal muscle. The major novel findings of this study were that: (1) LRP130 mRNA and protein expression were unaltered in response to acute and chronic SIT, respectively, and (2) there were several significant positive relationships between changes in LRP130, PGC‐1*α*, and SIRT3 protein content following SIT in human skeletal muscle.

### LRP130 expression following acute and chronic exercise

Whether changes in LRP130 expression occur in response to exercise in human skeletal muscle has yet to be determined, and this study is the first to examine LRP130 in response to an acute bout of exercise. Although swim training prevents reductions in *lrp130* mRNA expression caused by hindlimb immobilization in rats (Vechetti‐Junior et al. 2016), we found no change in *lrp130* mRNA expression 3 h after an acute bout of SIT in human skeletal muscle. Not surprisingly, we observed an increase in *pgc‐1α* mRNA expression, which increases in response to various types of acute aerobic exercise (Hellsten et al. 2007; Gibala et al. 2009; Edgett et al. 2013). In agreement with previous reports in rats (Hokari et al. 2010) and humans (Edgett et al. 2016), we also failed to observe an increase in *sirt3* mRNA expression 3 h post‐exercise in human skeletal muscle. These findings, in conjunction with our lack of observed change in *lrp130* mRNA expression, suggest that changes in *lrp130* and *sirt3* gene expression (similar to other genes linked to mitochondrial function) do not occur in response to acute SIT 3 h into the post‐exercise recovery period; however, if they do occur following acute exercise it is likely a delayed response (Pilegaard et al. 2003; Hellsten et al. 2007; Perry et al. 2010). Alternatively, we may have missed a change in *lrp130* mRNA expression prior to the 3 h post‐exercise time point examined in this study. Regardless, future studies should investigate whether other types (e.g., longer duration, steady state exercise), intensities, and durations of exercise regulate *lrp130* expression differently compared to SIT.

Another explanation as to why *lrp130* mRNA expression was unaltered in this study may be that participants were fed 1 h prior to the acute bout of SIT. This might have prevented a sufficient exercise‐induced decrease in muscle glycogen levels to stimulate changes in *lrp130* expression 3 h post‐exercise. Reductions in skeletal muscle glycogen content prior to an acute bout of exercise can potentiate the effect of exercise on mitochondrial gene expression in human skeletal muscle (Pilegaard et al. 2002), and nuclear localization of PGC‐1*α* in rat skeletal muscle (Philp et al. 2013), suggesting that *pgc‐1α* and *lrp130* expression may be modified by muscle glycogen levels. While we still observed an increase in *pgc‐1α* mRNA expression in response to SIT, it is possible that *lrp130* expression may be more sensitive to changes in skeletal muscle glycogen levels than *pgc‐1α*, or changes in *lrp130* expression may not be as robust as *pgc‐1α*, and thus we may have missed exercise‐induced changes in *lrp130* expression.

Although LRP130 protein expression is elevated in response to 7 days of swim training following hindlimb immobilization in rat skeletal muscle (Vechetti‐Junior et al. 2016), we observed no change in LRP130 protein expression following 3 and 6 weeks of SIT. The discrepancy between the findings of this study and those of Vechetti‐Junior et al. (2016) may be partly explained by species differences, and/or differences in basal levels of LRP130 expression due to the immobilization protocol implemented prior to training. However, given that LRP130 and PGC‐1*α* control each other's expression in cellular models (Cooper et al. 2006, 2008), and PGC‐1*α* protein content is reported to increase following various types of aerobic training in human skeletal muscle (Burgomaster et al. 2008; Perry et al. 2010; Ma et al. 2013), we hypothesized that LRP130 protein expression would increase in response to SIT. While it is somewhat unexpected that we also observed no change in PGC‐1*α* protein content following 3 or 6 weeks of SIT, others have reported similar results following 2 (Little et al. 2010; Vincent et al. 2015) and 4 weeks (Granata et al. 2016) of aerobic training. Thus, considering Vechetti‐Junior et al. (2016) observed an increase in LRP130 protein in association with elevated PGC‐1*α* in response to training, it is not necessarily surprising that LRP130 protein content did not change in response to SIT when PGC‐1*α* content was also unaltered, if these two proteins regulate each other's expression in human skeletal muscle.

### Relationship between SIRT3, LRP130, and PGC‐1α expression

An interesting finding of this study is that changes in LRP130 protein expression following SIT significantly correlated with changes in PGC‐1*α* expression. These results confirm observations that PGC‐1*α* and LRP130 protein were significantly correlated following 3 and 7 days of aerobic exercise in rats (Vechetti‐Junior et al. 2016), and suggests that the reciprocal regulation between LRP130 and PGC‐1*α* observed in liver cells and adipocytes (Cooper et al. 2006, 2008) may also be present in human skeletal muscle. Specifically, forced expression of LRP130 induces *pgc‐1α* mRNA expression in liver cells (Cooper et al. 2006) and adipocytes (Cooper et al. 2008), and increases PGC‐1*α* transcriptional activity (Cooper et al. 2006). As well, PGC‐1 double‐deficient brown adipocytes display dramatically lower levels of LRP130 mRNA and protein expression (Cooper et al. 2008). The addition of skeletal muscle‐specific LRP130 knockout and overexpression models would contribute greatly to this area, and future studies in humans should investigate the relationship between LRP130 and PGC‐1*α* expression following exercise training protocols known to upregulate PGC‐1*α* under different physiological stresses.

We also found a significant relationship between the change in SIRT3 and PGC‐1*α* protein content in response to 2 weeks of SIT (PRE to MID), but this relationship was not present following 6 weeks of training. Interestingly, in cell and animal models, SIRT3 has been implicated in the control of PGC‐1*α* expression (Shi et al. 2005; Palacios et al. 2009) and vice versa (Kong et al. 2010; Giralt et al. 2011; Vincent et al. 2015). Post‐training levels of SIRT3 and PGC‐1*α* protein content also significantly correlate in skeletal muscle of overweight adolescents (Vargas‐Ortiz et al. 2015). Accordingly, our results support the contention that SIRT3 and PGC‐1*α* may play a role in regulating each other's expression in human skeletal muscle.

## Conclusion

This is the first study to investigate changes in LRP130, and the relationships between changes in LRP130, PGC‐1*α*, and SIRT3 expression, in response to acute and chronic exercise in human skeletal muscle. LRP130 mRNA and protein expression were unaltered in response to acute and chronic SIT; however, individual changes in LRP130, PGC‐1*α*, and SIRT3 protein content were related following SIT. In agreement with previous studies investigating PGC‐1*α* (Konopka et al. 2010), our results indicate that changes in LRP130, PGC‐1*α*, and SIRT3 expression are not required for training‐induced increases in mitochondrial proteins and VO_2_peak. The finding that individual changes in LRP130, PGC‐1*α*, and SIRT3 protein content positively correlated at several time points in this study, in conjunction with previous reports in cell and animal models that indicate LRP130, PGC‐1*α*, and SIRT3 regulate each other's expression (Cooper et al. 2008; Palacios et al. 2009; Kong et al. 2010; Liu et al. 2014), suggests that the regulation of these proteins may be coordinated in human skeletal muscle. However, future studies should investigate larger doses of interval exercise, and/or longer duration steady state exercise known to increase PGC‐1*α* (Burgomaster et al. 2008; Perry et al. 2010; Ma et al. 2013) and SIRT3 (Johnson et al. 2015; Vargas‐Ortiz et al. 2015) protein content to identify if LRP130 expression is altered in response to other exercise protocols and to confirm these findings.

## Conflict of Interest

None declared.
